# Progress in Psychiatry

**Published:** 1902-10-18

**Authors:** 


*(To be continued.)*

### Progress in Psychiatry.

**Morphinism.**—Crothers[1] points out that the possibility of concealing its use for many years has made the morphia habit a most seductive vice. "Its use is prevalent in France and Germany, where entire villages are said to be filled with morphia-takers." From the amount of morphia consumed secretly, the number of morphia habitués in the United States is probably very large. The sale of morphia in some large cities of the United States indicates that not half of the amount sold is traceable to the legitimate demands of physicians and hospitals. Statistics of the sales of drug-stores show that a very large part of the total is bought by laymen. In one village in the centre of New York State a small drug-store sold 40 ounces a month. In a study of the morphia habit and of its prevalence among the medical profession in the United States, Crothers further states[2] that 10 per cent. out of a total of 3,244 physicians were estimated as either secret or open victims of the drug. Morphia is the drug most commonly used, whereas comparatively few take opium in the form of powder or of tincture (laudanum). "On the frontiers and in centres of great business excitement and nervous strain it is used more and more." The use of the hypodermic needle-syringe had added a fascination to the taking of morphia. The rapidity and certainty of the relief from pain, discomfort, or weariness which follows the injection leaves a mental impression not easily effaced. There is also a certain pleasure associated with the needle as thus used probably owing to belief that the power of the drug as an exhilarant and anodyne is increased by this method. This "hypodermic-needle mania" is likely to develop in neurotic persons who, when suffering from other diseases, have obtained marked relief from drugs given in this manner. In some parts of the States itinerant pedlars carry with them syringes and, for a consideration, treat persons who are suffering from pain or distress by injections of morphia. These peripatetic "doctors," so-called, have regular customers among the lower classes who receive injections daily. On the frontiers, where conditions of excitement and nerve strain result in exhaustion these morphia dealers are in great demand. Morphia taken by the hypodermic needle does not affect the stomach, hence its use is more popular.

**Stages of the Morphia Habit.**—In most cases the morphia habit exhibits three stages of development. The first stage is that in which the drug is taken to relieve pain or bodily suffering, such as colic, neuralgia, rheumatism, spasmodic asthma, dysmenorrhœa, or insomnia. The effect of the injection is usually satisfactory, as relief is speedily obtained. Later, when recurrences of pain take place, the remedy is again resorted to. In insomnia and in conditions of worry and anxiety attended with nervous exhaustion the effects produced by morphia are often very pleasant, as it not only relieves pain (anodyne action) but produces a feeling of general exhilaration and well-being (expansive action). The thought of becoming a victim addicted to the use of the drug and of becoming powerless to shake it off is not at this stage entertained by the patient, who takes it in strictly medicinal doses and only when needful. The second stage begins when the patient employs morphia for imaginary pains, and when he merely seeks pleasurable exhilaration for its own sake. After this the abandonment of the drug becomes difficult, and is followed by much discomfort and distress. Generally attempts are made from time to time to discontinue the habit, but in the majority of cases such attempts are unsuccessful. The patient now gets accustomed to use the drug at shorter and shorter intervals, and the craving which hitherto was only intermittent now becomes almost continuous. The third stage is marked by a continual craving for the drug, and the quantities consumed are large. Occasionally patients in this stage may be cured of the habit by the influence of powerful emotional disturbance (religious or moral influences). An example of this class is furnished by Dr. Crothers of a woman who was in the habit of taking six grains of morphia daily. She one day developed the conviction that this was the unpardonable sin. "She went to bed, and remained there for a week without drugs or treatment, and fully recovered." [3] In a second case the patient was a man taking five grains of morphia daily. One day he received a letter from a woman who had rejected his addresses some years before, offering to marry him on a certain specified date. He abandoned the use of the drug at once, and after remaining in bed two days went out restored, was married, and did not take the drug for at least two years afterwards. No further history was obtained of this case.[4] The insanity of morphinism is referred to by Dr. Robert Jones,[5] who gives an account of it as seen in eight patients, four males and four females, at the Claybury Asylum. One of these patients was a medical man, one a journalist, one a pianoforte maker, and one a coachman, while of the four female patients two were nurses and married. Three males and one female patient "contracted the habit from and through the advice of their unsuspecting medical attendants." Two of the four males practised the hypodermic injection of morphia, a third took morphia by the mouth and subcutaneously, and the fourth drank opium. Of the four females one took morphia by the mouth, another took it subcutaneously and by the mouth, a third took it in the form of chlorodyne, while in the fourth, owing to marked and unusual reserve, the method of administration could not be ascertained. One of the male patients took 20 grains of acetate of morphia daily, which he afterwards increased to 50 grains subcutaneously. Another stated that he took 4-grain doses hypodermically several times a day, and a third habitually took four ounces of laudanum per dose. Of the four women one took four to six grains of morphia as a dose. As regards the emotional tone of these patients it appeared that three of the males and two of the females showed melancholic depression, one male and one female were maniacal, and one female was the subject of delusional insanity. The general character of morphia takers was changed completely from the normal state. Anxiety, distrust, and depression were depicted in their pallid faces; they were restless, shifty, irritable, and unsociable. Volition was weakened, attention and application to work were impaired, and moral feebleness with neglect of home and of family duties and responsibilities were conspicuous features. The mental capacity finally grew enfeebled, until sequestration in an asylum or reformatory became necessary. The length of residence for treatment in an asylum would be less than four months, on an average. The symptoms to be guarded against during the period of enforced diminution of the drug were vomiting, diarrhœa, and cardiac failure or syncope. Sleeplessness and restlessness were also unfavourable symptoms during the first week of treatment, when the daily dosage of the drug was being rapidly "tapered" off. In all cases the patients were given an abundance of fluid diet—milk, beef-tea, tropon, plasmon—with occasional doses of brandy or whisky to combat collapse. Clifford Allbutt recommends the use of caffeine in the treatment of cardiac collapse which results from enforced abstinence, while Mattison of Brooklyn uses the bromides, codeine, and carnabis indica to produce sleep in insomnia resulting from the same enforced abstinence.

[1] Morphinism and Narcomanias from other Drugs, by T. D. Crothers, M.D., 1902. [2] Med. Rec., November 1899. [3] Crother's Morphinism and Narcomanias, p. 55. [4] Ibid. [5] Jour. of Mental Science, July 1902.

*(To be continued.)*

